# Percolation Phase Transition of Surface Air Temperature Networks under Attacks of El Ni*ñ*o/La Ni*ñ*a

**DOI:** 10.1038/srep26779

**Published:** 2016-05-26

**Authors:** Zhenghui Lu, Naiming Yuan, Zuntao Fu

**Affiliations:** 1 Lab for Climate and Ocean-Atmosphere Studies, Dept. of Atmospheric and Oceanic Sciences, School of Physics, Peking University, Beijing, 100871, China; 2 Department of Geography, Climatology, Climate Dynamics and Climate Change, Justus Liebig University Giessen, 35390 Giessen, Germany; 3 Key Laboratory of Regional Climate-Environment for Temperate East Asia, Institute of Atmospheric Physics, Chinese Academy of Sciences, Beijing, China

## Abstract

In this study, sea surface air temperature over the Pacific is constructed as a network, and the influences of sea surface temperature anomaly in the tropical central eastern Pacific (El Ni*ñ*o/La Ni*ñ*a) are regarded as a kind of natural attack on the network. The results show that El Ni*ñ*o/La Ni*ñ*a leads an abrupt percolation phase transition on the climate networks from stable to unstable or metastable phase state, corresponding to the fact that the climate condition changes from normal to abnormal significantly during El Ni*ñ*o/La Ni*ñ*a. By simulating three different forms of attacks on an idealized network, including Most connected Attack (MA), Localized Attack (LA) and Random Attack (RA), we found that both MA and LA lead to stepwise phase transitions, while RA leads to a second-order phase transition. It is found that most attacks due to El Ni*ñ*o/La Ni*ñ*a are close to the combination of MA and LA, and a percolation critical threshold *P*_*c*_ can be estimated to determine whether the percolation phase transition happens. Therefore, the findings in this study may renew our understandings of the influence of El Ni*ñ*o/La Ni*ñ*a on climate, and further help us in better predicting the subsequent events triggered by El Ni*ñ*o/La Ni*ñ*a.

El Ni*ñ*o/La Ni*ñ*a, characterized by anomalous warming/cooling in the tropical central eastern Pacific, is one of the most important ocean-atmosphere coupled phenomena in climate system. It has great influences on climate, which may further cause natural disasters like flood and drought^1^,^2^,^3^,^4^,^5^,^6^. The climate during El Ni*ñ*o/La Ni*ñ*a is quite different from that during normal periods. Although a lot of studies about El Ni*ñ*o/La Ni*ñ*a have been done, there are still many problems unsolved, such as the prediction of El Ni*ñ*o/La Ni*ñ*a^7^,^8^,^9^, impact of El Ni*ñ*o/La Ni*ñ*a on climate^10^,^11^, distinction of different types of El Ni*ñ*o/La Ni*ñ*a^12^,^13^,^14^, and so on. In order to solve these problems, or modestly speaking, to better understand El Ni*ñ*o/La Ni*ñ*a, it is necessary to develop new methods and from new perspectives to study El Ni*ñ*o/La Ni*ñ*a.

Recently, more and more studies have applied the concept of complex networks to investigate climate system. It is called climate network, where different regions of the world are represented as nodes which communicate with each other by exchanging heat, material, and even forces. These interactions can be represented by links, which are quantified by measuring the similarity between time series of corresponding individual nodes. Any two nodes are connected if the similarity, or link strength 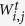 (see “Method” section) of a specific variable measured at the two nodes is beyond a threshold^15^. The concept of climate network effectively introduced the vast framework of network analysis to climate science and triggered plenty of research activities in this area, such as making climate prediction^16^,^17^,^18^,^19^,^20^, evaluating effects of natural modes of climate variability on network properties^21^,^22^,^23^,^24^,^25^,^26^,^27^,^28^, and so on.

Percolation theory is one of the most interesting findings in complex networks but has never been applied to study climate network^29^,^30^,^31^,^32^,^33^,^34^,^35^,^36^,^37^,^38^,^39^,^40^,^41^,^42^,^43^,^44^,^45^. The theory indicates the existence of a critical probability *P*_*c*_, such that above *P*_*c*_ the network is divided into isolated clusters, while below *P*_*c*_ a giant cluster still spans the entire network. The critical probability *P*_*c*_, called as the percolation threshold, can be defined as the fraction of node removal, which in other words, means the percentage of node that the links have been cutoff from the entire network. This kind of node removal can be considered as an attack on network, and the phenomenon is markedly similar to a percolation phase transition. Once the phase state converts from stable to unstable or metastable, the network will be broken totally or partly. Different phase states mean different physical conditions. In real world, many complex systems like road and railway networks, electrical power networks, internet networks, usually can be damaged by malicious attacks or natural disasters, such as power blackout and earthquake^29^,^32^,^33^,^34^,^36^. Analogously, in climate system, the network may also converts its state under the influences of some big climate events, such as El Ni*ñ*o/La Ni*ñ*a. From percolation theory’s point of view, we can consider the big climate events as attacks on the climate network. Since percolation theory could distinguish different network states and further monitor transitions of different states, we believe it should be also useful in climate research, and may provide us with a new perspective on the climate diagnosis, monitoring and forecasting. Hence, in this study, we will discuss the percolation phase transition in the climate networks. Considering El Ni*ñ*o/La Ni*ñ*a is one the most important phenomenon in climate system, we will mainly focus on the network transition under the attack of El Ni*ñ*o/La Ni*ñ*a.

This paper is organized as following: In the “Results” section, we first constructed a network by using the surface air temperature over Pacific and studied the influences of El Ni*ñ*o/La Ni*ñ*a. To check whether there is a phase transition, and according to which way the state is converted, we further established an idealized network and simulated the transitions under different kinds of attacks. With all the findings, a detailed discussion is made in the “Conclusion and Discussion” section, and a brief description of the data and methods are provided in the end of this paper.

## Results

### Attacks of El Ni*ñ*o/La Ni*ñ*a on surface air temperature network

**Figure 1:**
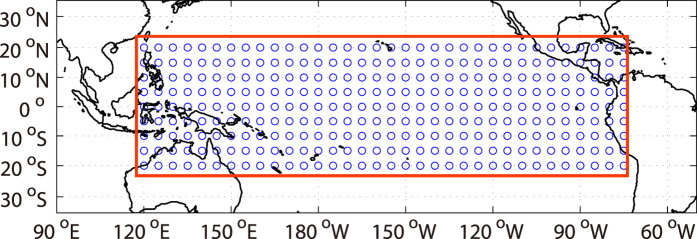
Research domain of this study. With in the red box, 306 nodes with resolution of 5° × 5° are selected and the corresponding surface air temperatures are constructed as a climate network. The figure is generated by using Matlab (version R2012a, http://www.mathworks.com/pl_homepage).

In this study, the surface air temperatures over the spatial domain 120°*E* to 285°*W* and 20°*N* to 20°*S* are constructed as a network. As shown in Fig. 1, the nodes in the network have a resolution of 5° × 5°, and they are marked with numbers from 1 to 306 according to the sequence from west to east and from north to south. Because the atmosphere above ocean can easily be heated/cooled by the sea surface temperature anomaly (SSTA), which means the links in the surface air temperature network can easily be influenced^26^,^27^. To quantify in what extent the SSTA, such as the El Ni*ñ*o/La Ni*ñ*a, can affect the network, we use two quantities, as shown below:

1. The total degrees of connection (*D*_*T*_). As described in “Method” section, if the link strength between two nodes exceeds a threshold *Q*, we consider the two nodes are connected, and there will be one degree of connection counted. By summing the degrees over all nodes, we obtain the total degree of connection *D*_*T*_.
2. The intensity of attack (*P*). If a node is not connected with any other nodes, we consider the node as isolated node. The intensity of attack is then defined as the fraction of isolated nodes over the total nodes.

**Figure 2:**
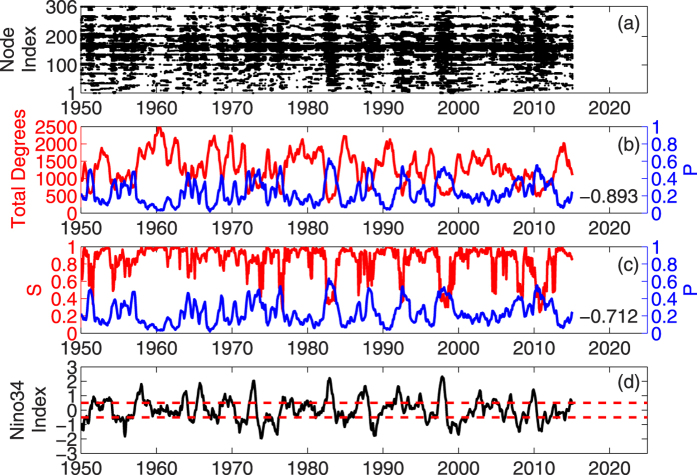
Temporal variation of several properties of the surface air temperature network. (**a**) shows the distribution of isolated nodes as a function of time and the node index (1~306). The black dots represent the isolated nodes. (**b**) shows the temporal variation of the total connection degree *D*_*T*_ (red) and the intensity of attack *P* (blue). The two curves have very significant negative correlations (*r* = −0.893). (**c**) shows the giant component size *S* (red) as well as the intensity of attack *P* (blue). Again, the two curves have very significant negative correlations (*r* = −0.712). (**d**) shows the monthly Ni*ñ*o3.4 index (black). The red dashed lines represent +0.5 and −0.5, respectively.

If as expected, the effects of El Ni*ñ*o/La Ni*ñ*a on the surface air temperature network can be considered as a kind of attack, which will damage the connections of the nodes in the network, then during the El Ni*ñ*o/La Ni*ñ*a events, the total degree of connection *D*_*T*_ should decrease, and the fraction of isolated nodes (Intensity of attack, *P*) should increase. See Fig. 2, we indeed find the expected varying patterns, which correspond to Nino3.4 index very well. Figure 2a shows the distribution of isolated nodes as a function of time (x-axis) and the node index (1~306, y-axis). The black dots represent the isolated nodes. Figure 2b shows the temporal variation of total connection degree *D*_*T*_ (red) and intensity of attack *P* (blue), while Fig. 2d shows the Nino3.4 index. As one can see, during El Ni*ñ*o/La Ni*ñ*a events (when the Nino3.4 index is larger/smaller than +0.5/−0.5, see Fig. 2d), there will be much more nodes isolated (Fig. 2a), much stronger *P* (Fig. 2b), and much lower total connection degrees *D*_*T*_ (Fig. 2b). Therefore, we believe the sea surface temperature anomaly (El Ni*ñ*o/La Ni*ñ*a) indeed can attack the surface air temperature network, and isolate nodes from the network.

**Figure 3:**
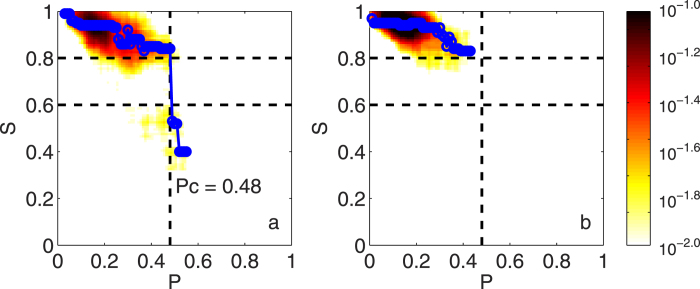
Study of the percolation phase transition in the surface air temperature network. Two groups are classified according to the Nino3.4 index. For the group of normal cases (**b**), the giant component size *S* from nearly all the time points are above 0.8. While for the anomalous cases (**a**), *S* drops abruptly as soon as the attack intensity *P* exceeds a threshold (around 0.48), which indicates phase transition in the surface air temperature network. The color shown in this figure represents the probability for a given case (time point) to have the corresponding *S* and *P*. The blue circles represent the maximum values of the probability in each interval with a bin size of 0.01 from *P* = 0 to 1, which are connected by the blue lines. The vertical black dashed line represents 0.48.

Since nodes can be isolated under the attack of El Ni*ñ*o/La Ni*ñ*a, and the network can be divided into several clusters (cluster, a part of the network, where any two nodes can be connected with at least one path), another question comes out: will there be a phase transition in the network? Or in other words, will there be a threshold that above the threshold the state of the network will convert abruptly? To address this question, another quantity, the giant component size *S*, is used in our study. As described in “Method” section, the giant component size *S* is defined as the ratio of the number of nodes in the largest cluster and the number of total connected nodes. It is an indicator from percolation theory, and can be used to determine whether the phase of the network has been changed significantly. As shown in Fig. 2c (the red line), in surface air temperature network, the giant component size *S* is significantly correlated with the attack intensity *P* (Corr = −0.712), and *S* falls abruptly when *P* is increasing. This indicates the percolation phase transition may appear in the surface air temperature network. To better show how the network reacts under the attack of El Ni*ñ*o/La Ni*ñ*a, we further classified all the considered time points (1948~2015) into two groups according to the Nino3.4 index. If the Nino3.4 index is larger (or smaller) than 0.5 (or −0.5), we name them as anomalous cases, otherwise, we name them as normal cases. By studying how the giant component size *S* varies with different *P* in the two groups, we obtain Fig. 3, where significant differences can be found. During normal period (−0.5 < Nino3.4 index < 0.5, see Fig. 3b), the giant component size *S* from nearly all the cases are above 0.8. While during anomalous period (Nino3.4 index > 0.5, or Nino3.4 index < −0.5, see Fig. 3a), one can easily find the giant component size *S* drops abruptly from a high level (above 0.8) to a low level (below 0.6) once the attack intensity *P* is larger than 0.48. According to percolation theory, this kind of abrupt jumping, called abrupt percolation phase transition, is similar to the first-order phase transition, with the giant component size *S* suddenly jumping from a relatively high value to a relatively low value at a transition point (i.e. critical threshold), which indicates the phase state of the network converts from stable to unstable or metastable abruptly. *P* = 0.48 seems to be the critical threshold. For the cases during normal period (Fig. 3b), since the attack of the sea surface temperature anomaly (SSTA) in the tropical central eastern Pacific is not strong enough (*P* < 0.48), no abrupt changes of the network happened. However, for some cases during anomalous period (Fig. 3a), the attack of the SSTA can be strong enough (*P* > 0.48) to lead to the phase transition. Therefore, from Fig. 3, we may conclude that once the attack intensity of El Ni*ñ*o/La Ni*ñ*a exceeds a critical threshold (*P*_*c*_ = 0.48), an abrupt phase transition of the above surface air temperature network can be expected.

### Simulation of the El Ni*ñ*o/La Ni*ñ*a attack by using idealized network

**Figure 4:**
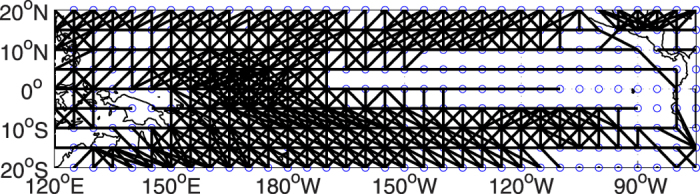
The idealized network. Black lines means connections. From this figure, if two nodes are linked by a black line, we say the two nodes are set as connected in the idealized network. The figure is generated by using Matlab (version R2012a, http://www.mathworks.com/pl_homepage).

To further study the influences of El Ni*ñ*o/La Ni*ñ*a on the surface air temperature network, especially to study in which way the nodes in the network are isolated under the attack of El Ni*ñ*o/La Ni*ñ*a, we in this section constructed an idealized network. We first made a statistic on the connections between each pair of nodes in the network. For the pair of nodes where connection frequently appeared (see Supplementary Fig. 2, we have defined a threshold to determine whether the connection frequently appears or not), we set there is a connection between them in the idealized network. As shown in Fig. 4, in this way, an artificial network is constructed, which may represent the “background climatology” of the network. Before further studies, we checked the ability of the idealized network in simulating the reactions under attacks of El Ni*ñ*o/La Ni*ñ*a in real world network. As shown in Supplementary Fig. 3, under attacks of El Ni*ñ*o/La Ni*ñ*a, both the total degrees of connection *D*_*T*_ and the giant component size *S* have shown very similar temporal patterns as the the results found from the real network. Therefore, this idealized network is reasonable and appropriate for further simulating the reactions under different kinds of attacks.

**Figure 5:**
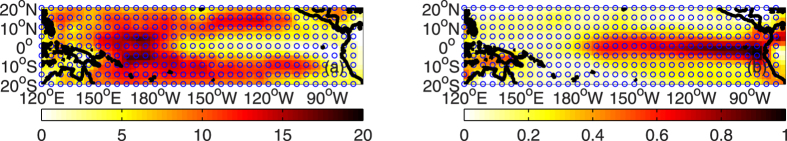
Spatial distributions of node degrees *K*_*i*_ and node vulnerabilities *F*_*i*_. (**a**) shows the spatial distribution of *K*_*i*_ in the idealized network (see Fig. 4). A node with red color means it has more connections (higher *K*) than a node with yellow color. Therefore, this figure corresponds to the Most connected Attack (MA), which removes nodes according to the decreasing sequence of node degree *K*_*i*_. (**b**) shows the spatial distribution of *F*_*i*_. A node with red color means it is more vulnerable (higher *F*) than a node with yellow color. Therefore, this figure corresponds to the Localized Attack (LA), which removes nodes according to the decreasing sequence of node vulnerability *F*_*i*_. These two figures are generated by using Matlab (version R2012a, http://www.mathworks.com/pl_homepage).

**Figure 6:**
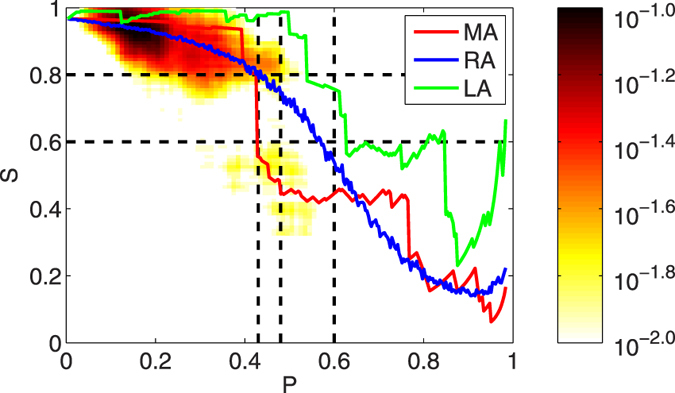
Simulation results of percolation phase transition in the idealized network. Results of different kinds of attacks are shown by curves with different colors. MA: red, RA: blue, and LA: green. The areas with different colors are the results from Fig. 3a. As one can see, both MA and LA can lead to stepwise phase transitions, and the results of MA fit better to the results from real networks. The colored area mainly concentrates on two phase states, which indicates a phase transition indeed can happen under the attack of El Ni*ñ*o/La Ni*ñ*a. The vertical black dashed lines represent 0.43, 0.48 and 0.6, respectively.

To the end of this section, we will study the reactions of the idealized network under different kinds of attacks. Since El Ni*ñ*o/La Ni*ñ*a basically occurs in the tropical central eastern Pacific, their attacks on the surface air temperature network of course are more likely to appear in this region, as shown in Fig. 5b. Therefore, the first kind of attack we use is the Localized attack (LA)^46^, where the nodes are isolated according to their vulnerability to the attacks of El Ni*ñ*o/La Ni*ñ*a, see Fig. 5b. Besides LA, another two kinds of attack are also employed. One is the Most connected Attack (MA), and the other is the Random Attack (RA)^31^,^35^. MA means the nodes will be isolated according to the connection degrees. See Figs 4 and 5a, the node with the most connections will be removed first, and so on. While RA means the nodes will be isolated randomly. By removing nodes, in other words, by increasing the intensity of attacks *P*, we can calculate the corresponding giant component size *S* from the idealized network. Figure 6 shows the results for LA (green), MA (red), and RA (blue) respectively. Moreover, we spend 100 times in simulating the results of RA and take the average. As one can see, both LA and MA can lead to a stepwise phase transition, while RA leads to a second-order phase transition^32^,^42^. Since as shown in Fig. 2, the intensity of attacks *P* due to El Ni*ñ*o/La Ni*ñ*a is below 0.6, we thus only focus on the *P* interval between 0 and 0.6. For MA, interestingly there exits an obvious critical threshold *P*_*c*_ at around 0.43, which is close to the critical threshold found in Fig. 3. For LA, one can also see a critical threshold, but is around *P* = 0.5 and *P* = 0.6. By comparing the percolation phase transition simulated from the idealized network with the results calculated from the real network (Fig. 3), it is easy to find that the results from MA have the best simulation, which means the attacks of El Ni*ñ*o/La Ni*ñ*a on the surface air temperature network may mainly follow the way of MA. That is, the nodes with the most connections may be isolated earlier than the nodes with less connections. However, we like to note that we are studying the influences of El Ni*ñ*o/La Ni*ñ*a, there should be no doubt that the nodes in the key region (tropical central eastern Pacific) are more vulnerable. As shown in Fig. 5b, the nodes in the key region are more frequently isolated under the attacks of El Ni*ñ*o/La Ni*ñ*a. Therefore, besides MA, the influence of El Ni*ñ*o/La Ni*ñ*a on the surface air temperature network should also to some extent follow the way of LA. In other words, the El Ni*ñ*o/La Ni*ñ*a may prefer two ways (MA and LA) to attack the above air temperature network. When El Ni*ñ*o/La Ni*ñ*a happens, both the local nodes and the nodes with the most connections are more likely to be isolated. But as shown in Fig. 6, it is not easy for LA to reach a critical threshold (*P*_*c*_ is between 0.5 and 0.6), therefore, as the nodes are removed during an El Ni*ñ*o/La Ni*ñ*a event, the state of the network is more likely to convert following the way of MA, where the *P*_*c*_ is much smaller (around 0.43).

## Conclusion and Discussion

In this work, the influence of El Ni*ñ*o/La Ni*ñ*a are studied from a new perspective: network. We consider the surface air temperature field over Pacific as a network, and the influence of El Ni*ñ*o/La Ni*ñ*a as attacks on the network. By measuring quantities such as the total degrees of connection *D*_*T*_ and the intensity of attack *P*, we find the surface air temperature network will be influenced in terms of isolating nodes under the attacks of El Ni*ñ*o/La Ni*ñ*a. By further studying the giant component size *S*, which is a quantity from the percolation theory, we find that there exists a critical threshold *P*_*c*_, above which the network will convert its state abruptly. From the idealized network, we studied the network reactions under different kinds of attacks, and find that the most possible ways for the network to be influenced under the attacks of El Ni*ñ*o/La Ni*ñ*a, is LA (Localized Attack) and MA (Most connected Attack). This means, the nodes which located in the relevant region (tropical central eastern Pacific) and the nodes with the most connections will more likely be isolated. With the increasing of attack intensity *P*, more and more nodes will be isolated. At a critical point (*P*_*c*_ around 0.48), the state of the surface air temperature network will change abruptly from stable to unstable or metastable.

This work provides us with a new view to understand the influence of El Ni*ñ*o/La Ni*ñ*a on climate. Suppose we consider the climate state during normal periods as stable phase states. When El Ni*ñ*o/La Ni*ñ*a happens, the influence of sea surface temperature anomaly will be first transferred into the above atmosphere, where the former connections (during normal periods) may be cutoff and new connections may be established. As a result, the atmosphere state may be changed, or in other words, the atmosphere will be coupled differently with the ocean. With the new state, the atmosphere will further transfer the influence of El Ni*ñ*o/La Ni*ñ*a to other remote regions, such as East Asia Monsoon region, etc. This is the well known way how El Ni*ñ*o/La Ni*ñ*a is teleconnected with remote regions, and the atmosphere plays a role as a bridge (Atmospheric Bridge). In this study, by using the concept of network and the theory of percolation, we studied how the El Ni*ñ*o/La Ni*ñ*a events can influence the surface air temperature network. We focused on the former connections from the normal periods (without El Ni*ñ*o/La Ni*ñ*a) and revealed how the former connections may be attacked by El Ni*ñ*o/La Ni*ñ*a. The results show that:

1. Not all sea surface temperature anomalies in the tropical central eastern Pacific can induce the transition of states in the above atmosphere. The intensity of attack should exceed the critical threshold *P*_*c*_ (around 0.48).
2. When the influence is strong enough, the state of the above atmosphere will convert abruptly, not gradually.

In this study, we classified the considered time points into two groups according to the Nino3.4 index. For the group of normal cases (−0.5 < Nino3.4 index < 0.5), the attack intensity *P* is not strong enough and no abrupt changes of *S* is found. While for the group of anomalous cases (Nino3.4 index > 0.5, or Nino3.4 index < −0.5), the *P* can be larger than 0.48, and abrupt phase transitions are found. Therefore, we need to note that the traditional definition of El Ni*ñ*o/La Ni*ñ*a is reasonable and indeed useful in monitoring El Ni*ñ*o/La Ni*ñ*a events. However, we would like to emphasize that not all the cases when Nino3.4 index > 0.5, or Nino3.4 index < −0.5, can result in a phase transition of the surface air temperature network. As discussed above, to better evaluate the influences of El Ni*ñ*o/La Ni*ñ*a, or even predict the subsequent events trigged by El Ni*ñ*o/La Ni*ñ*a, one is suggested to first measure the intensity of attack *P* to check whether the above atmosphere has changed its state. If *P* is larger than *P*_*c*_, the phase of the surface air temperature network will be changed abruptly, and the influence of El Ni*ñ*o/La Ni*ñ*a may further be transferred to remote regions. Otherwise, one has to consider the possible effects of El Ni*ñ*o/La Ni*ñ*a more modestly.

Obviously, from this study we can see *P*_*c*_ is the key quantity, which is not only important for determining of percolation phase transition in the network, but also helpful in improving our climate prediction skills. In this work, we found *P*_*c*_ is around 0.48, but we need to admit that more detailed researches are still necessary. For instance, we need to check if the *P*_*c*_ value remains unchanged for El Ni*ñ*o Modoki, whether *P*_*c*_ = 0.48 is universal for all El Ni*ñ*o/La Ni*ñ*a events, and so on. Furthermore, we also need to note that, network theory and percolation theory are new in climate studies. Besides the research on El Ni*ñ*o/La Ni*ñ*a, other explorations from this new perspective are also needed in the future.

## Data and Methods

### Data

In this study, the daily surface air temperature from NCEP/NCAR reanalysis 1 project for years 1948–2015 are used^47^. The data are downloaded from the National Oceanic & Atmospheric Administration (NOAA, http://www.esrl.noaa.gov/psd/data/gridded/data.ncep.reanalysis.surface.html). Spatial domain between 120°*E* and 285°*W*, 20°*N* and 20°*S* are selected for the analysis, and the horizontal resolution is 5° × 5° (see Fig. 1, from the original dataset which has horizontal resolution of 2.5° × 2.5°, every other grid point is selected as a node in Fig. 1). Each node is marked with numbers from 1 to 306 as node index, according to the sequence from west to east and from north to south. Besides the surface air temperature, the monthly Nino3.4 Sea Surface Temperature Anomaly (Nino34 index) is also used as an indicator of El Ni*ñ*o/La Ni*ñ*a events (see Fig. 2d). The index is also downloaded from NOAA (http://www.esrl.noaa.gov/psd/data/climateindices/).

## Methods

### Surface air temperature Network

We employ the nonlinear synchronization measure to construct a network^23^,^26^,^27^. For each node in Fig. 1, we first extract anomaly values by subtracting long-term mean annual cycle (leap days are removed), *T*_*k*_(*d*), where *k* is the node index and *d* is the calendar date. Then we compute, for every 30th day *t* in the considered time span between January 1950 and August 2015, the time-delayed cross-correlation coefficients for each pair of nodes *i* and *j* over 365 days before *t*, with time lags *τ* between −200 days and 200 days. The result is denoted by 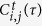. Finally we determine, for each time point *t*, the maximum, the mean and the standard deviation of the absolute values of the cross-correlation coefficients 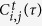, and further define the link strength as^23^,^26^,^27^


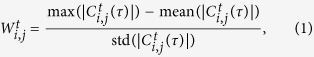


A pair of nodes is considered as connected if their link strength is above a threshold *Q*^48^,^49^ (for confidence level of 99%, *Q* = 0.57. See the Supplementary Figure 1), and one degree of connection is counted. Otherwise, we say there is no connection between the two nodes under a given confidence level. By using Heaviside function, we can represent this definition as


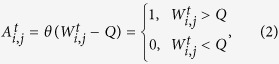


and the degrees of node *i* at time *t* is thus represented as,


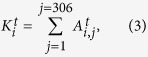


where *i* and *j* are both the node indexes from 1 to 306. By summing the degrees of all the nodes, we will further obtain the total degrees of connection *D*_*T*_,


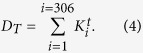


If at a given time point *t*, node *i* has no connections with any other nodes, 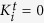, we name it as an isolated node. To better describing this concept, we further defined a new quantity as 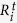,


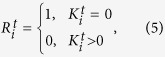


where *i* is the node index from 1 to 306. If the node *i* at time point *t* is an isolated node, we set 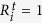, otherwise, 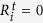. See Fig. 2a, the distribution of isolated nodes over time is shown as black dots.

### Intensity of attacks

In percolation theory, intensity of attacks is usually defined as the fraction of nodes removed from original network. When a node is removed, all links between this node and other nodes will be cut off and this node will become an isolated node. Analogously, in the surface air temperature network, we consider the isolated nodes as the result of attacks such as El Ni*ñ*o/La Ni*ñ*a, and define the intensity of attacks at time point *t* as,


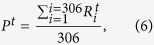


where 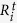 is quantity defined in equation (5), and *P*^*t*^ represents the fraction of the isolated nodes, which as defined is the intensity of attacks at time point *t*. As shown in Fig. 2b,c, the blue curve shows the intensity of attacks over time.

### Node Vulnerability

To quantify the vulnerability of a given node under attacks, we defined another quantity as,


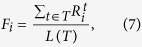


where *L*(*T*) represents the length of a given time period (the total time points), and *F*_*i*_ is the fraction of the time points when node *i* is isolated over the total time points. See Fig. 5b, the spatial distribution of *F*_*i*_ is show with different colors.

### Giant component size

In percolation theory, giant component size is used to measure fragmentation and functionality of network, and indicate the phase state of network. To calculate the giant component size, one needs to first find the largest cluster, where i) any two nodes can be connected with at least one path, and ii) the number of nodes is the highest. Then the giant component size at time point *t* can be defined as,


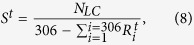


where *N*_*LC*_ is the number of nodes in the largest cluster, and *S*^*t*^ represents the giant component size at time point *t*. See Fig. 2c, the giant component size *S* are shown as the red curve. It is worth to note that besides the *S* defined as equation (8), there is another widely used definition, where the giant component size are defined as the ratio of the number of nodes in the largest cluster and the number of the total nodes^50^. By using this definition, we find similar results as what we show in this paper, therefore the conclusions in our research stay unchanged.

### Idealized network

In meteorology, composite analysis is widely used to determine a background field during a specific time period. In this study, we borrow this idea to construct a idealized network. Suppose there exists an idealized network which represents the state of climate network under slight or even no attacks from El Ni*ñ*o/La Ni*ñ*a, but when El Ni*ñ*o/La Ni*ñ*a happens, it can react similarly as the real network. To establish such a network, we need to first find the time points when no (or only very slight) attack happens. By setting *S*^*t*^ > 0.98, we can select the time points out as *T*′,


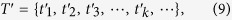


where *t*′_*k*_ represents the time point when there is no (or very slight) attack and the giant component size *S* > 0.98. Then we calculate the frequency of occurrence for each connection (e.g., connection between node *i* and node *j*, we can also name it as edge between *i* and *j*) in the selected time points *T*′,


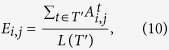


where *L*(*T*′) is the number of time points in *T*′, and *E*_*i*,*j*_ represents the frequency of occurrence of the connection between node *i* and node *j*. If *E*_*i*,*j*_ is higher than a threshold *E*, which indicates a high possibility that the two nodes are connected, we therefore will set the two nodes connected in the idealized network, as *C*_*i*,*j*_ = 1. Otherwise, we set the two nodes separated, as *C*_*i*,*j*_ = 0. With the help of Heaviside function, *C*_*i*,*j*_ can be summarized as,


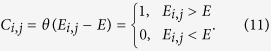


The threshold *E* should satisfy two conditions. First, with an appropriate *E*, the established idealized network should have a big giant component size, *S* > 0.98. Second, an appropriate threshold *E* should make sure that the total degrees of connection *D*_*T*_ of the idealized network does not exceed the range of *D*_*T*_ of the real networks over the selected time points *T*′. As introduced in Supplementary Fig. 2, we in this study choose *E* = 0.29. According to the above procedures, the idealized network is constructed, as shown in Fig. 4.

## Additional Information

**How to cite this article**: Lu, Z. *et al*. Percolation Phase Transition of Surface Air Temperature Networks under Attacks of El Ni*ñ*o/La Ni*ñ*a. *Sci. Rep.* **6**, 26779; doi: 10.1038/srep26779 (2016).

## Supplementary Material

Supplementary Information
