# Supplementary material for: Percolation Phase Transition of Surface Air Temperature Networks under Attacks of El Niño/La Niña
Source: Sci Rep. 2016 May 26;6:26779. doi: 10.1038/srep26779 (PMC4880929; doi:10.1038/srep26779)
Supplement: Supplementary Information [file srep26779-s1.pdf]

Percolation Phase Transition of Surface Air Temperature Networks under Attacks of El Niño/La Niña

(Supplementary Materials)

Zhenghui Lu[1] Naiming Yuan[2] Zuntao Fu[1] *

1 Lab for Climate and Ocean-Atmosphere Studies, Dept. of Atmospheric and Oceanic Sciences, School of Physics, Peking University, Beijing, 100871, China

2 Key Laboratory of Regional Climate-Environment for Temperate East Asia, Institute of Atmospheric Physics, Chinese Academy of Sciences, Beijing, China

*Correspondence author: Zuntao Fu, Lab for Climate and Ocean-Atmosphere Studies, Dept. of Atmospheric and Oceanic Sciences, School of Physics, Peking University, Beijing, 100871, China. Email: fuzt@pku.edu.cn; Tel:86-010-62767184

List of Figures:

Supplementary Figure 1: Probability density functions (PDF) of link strength for shuffled network ($W^t_{s;i,j}$) and for real network ($W^t_{i,j}$).

Supplementary Figure 2: Giant component size S and total degrees D_T of the idealized network constructed under different threshold E.

Supplementary Figure 3: Total degrees D_T and giant component size S of the real network and the idealized network.

Supplementary materials for determining connections between nodes. For weighted network, according to equation (1) in our manuscript, one can always calculate a link strength $W^t_{i,j}$ between node i and node j, at time point t. But whether the link has true physical meanings, or just spurious result due to random effect, it is important to determine a threshold Q. Similar to the studies performed by [1, 2], in our study, we first shuffled the original time series at each node randomly, and then calculated the link strength $W^t_{s;i,j}$ of each pair of nodes as what we did for the original network (see the "Method" section, equation (1) in our manuscript). By comparing the probability density functions (PDF) of link strength $W^t_{i,j}$ (from the real network) and $W^t_{s;i,j}$ (from the shuffled network), where t covers all the years from 1950 to 2015, including El Niño, La Niña and normal years, as shown in Supplementary Fig.1, we can determine the threshold Q, above which a true connection between the two nodes i and j can be confirmed. In our study, we find $Q = 0.57$, which is reasonable because of the following two reasons: i) it satisfies the significance level of 0.01 for $W^t_{s;i,j}$ (see Supplementary Fig.1a); ii) it is the inflexion point in the PDF of $W^t_{i,j}$ for the real network (see Supplementary Fig.1b).

Supplementary materials for the idealized network. Building an idealized network is another important part of our research. Although we have introduced the method of how to build the idealized network in the "Method" section of the manuscript, it is still necessary to show more details. As mentioned in the manuscript, only the time points when there is no (or very slight) attack ($S^t > 0.98$) are selected and the frequency of occurrence for each connection $E_{i,j}$ is calculated according to equation (10) in the manuscript. By using a threshold E, we can then determine whether there should be

a connection set up between node i and j in the idealized network (see equation (11)). Accordingly, the idealized network will be established. For different threshold E, we will have different idealized network built. Therefore, it is important to use an appropriate threshold E. In our work, the giant component size S and the total degrees of connection D_T are used as judgments for the selection of E. See the blue curve and the green curve shown in Supplementary Fig.2, with the threshold E increases, both S and D_T decrease. However, since the idealized network are established based on the statistics of the real network when $S^t > 0.98$, it should also satisfy the condition $S > 0.98$, which means $E \leq 0.29$. On the other hand, since the maximum D_T of the real network do not exceed 2650, we would better to control the total degrees of the idealized network below 2650, which means $E \geq 0.23$. As a result, we set the lower and upper limits of E as 0.23 and 0.29, respectively. In our work, we choose $E = 0.29$ as the threshold and the idealized network is determined accordingly. See Fig.4 in the manuscript.

Before we use this idealized network for further researches, it is necessary to test its ability in simulating the reactions of real network to the attacks of El Niño/La Niña. From the real network, we can calculate whether a node i, at time point t, is isolated or not (please refer to the quantity R_i^t, equation (5) in the manuscript). Suppose the idealized network received the same attacks as the real network, as shown in the following equation,

$$C_{i,j}^t = C_{i,j} * \theta(-R_i^t - R_j^t), \tag{1}$$

if the node i or node j are isolated in the real network at time point t, we set the connection between i and j in the idealized network is removed, which means the idealized network

suffers the same attacks as the real network. $\theta(x)$ is the Heaviside function. In this way, we can compare the idealized network with the real network, by using two quantities: total degrees of connection D_T and the giant component size S. As shown in Supplementary Fig.3, both D_T and S calculated from the idealized and real network show high similarity with high correlation coefficient 0.904 and 0.610, respectively. Therefore, the idealized network indeed can simulate the reactions of real network under different kinds of attacks.

References

[1] Wang, Y. et al. Dominant Imprint of Rossby Waves in the Climate Network, *Phys. Rev. Lett.*, **111**, 138501 (2013).

[2] Guez, O., Gozolchiani, A., and Havlin, S. Influence of autocorrelation on the topology of the climate network, *Phys. Rev. E*, **90**, 062814 (2014).

Figure Legends

Supplementary Figure 1: **Probability density functions (PDF) of link strength for shuffled network ($W^t_{s;i,j}$) and for real network ($W^t_{i,j}$).** a) shows the PDF of link strength calculated from the randomly shuffled network. b) shows the PDF of link strength calculated from the real network. The dashed line shows the threshold of 5.7, which in a) represents the significance level of 0.01, and in b) represents the point of inflexion. t covers all the years from 1950 to 2015.

Supplementary Figure 2: **Giant component size S and total degrees D_T of the idealized network constructed under different threshold E.** The blue curve represents S, while the green curve represents D_T. The right vertical dashed line represents the upper limit of $E = 0.29$ with S larger than 0.98. The right vertical dashed line represents the lower limit of $E = 0.23$ when D_T is smaller than 2650 (he horizontal dashed line).

Supplementary Figure 3: **Total degrees D_T and giant component size S of the real network and the idealized network.** a) shows the total degrees of connection D_T, and b) shows the giant component size S. The black curves are the results calculated from the real network, while the red curves are the results simulated from the idealized network. As one can see, the black and the red curves have similar patterns with very high correlation coefficient: 0.904 for D_T, 0.610 for S. Therefore, the idealized network indeed has the ability in simulating the reactions of real network to the attacks of El Niño/La Niña.

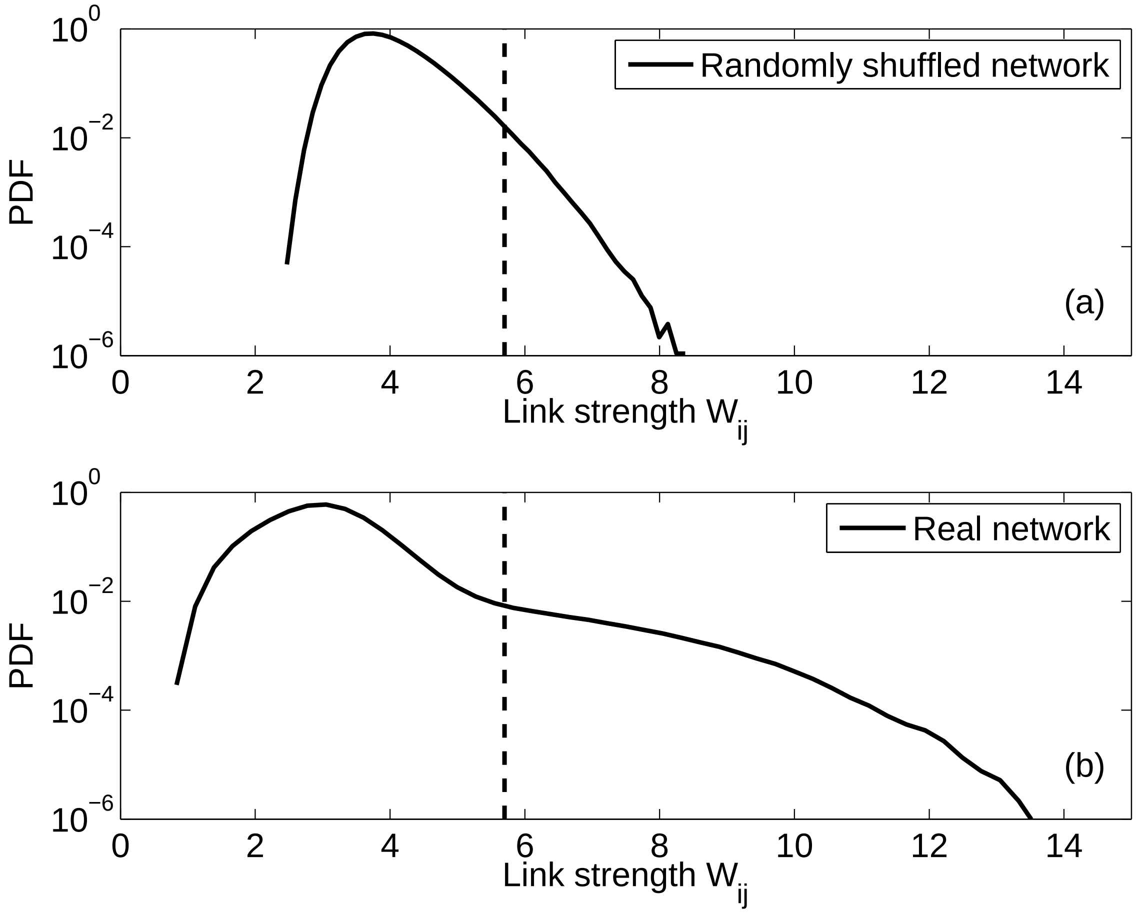

Figure 1: **Probability density functions (PDF) of link strength for shuffled network ($W^t_{s;i,j}$) and for real network ($W^t_{i,j}$).** a) shows the PDF of link strength calculated from the randomly shuffled network. b) shows the PDF of link strength calculated from the real network. The dashed line shows the threshold of 5.7, which in a) represents the significance level of 0.01, and in b) represents the point of inflexion. t covers all the years from 1950 to 2015.

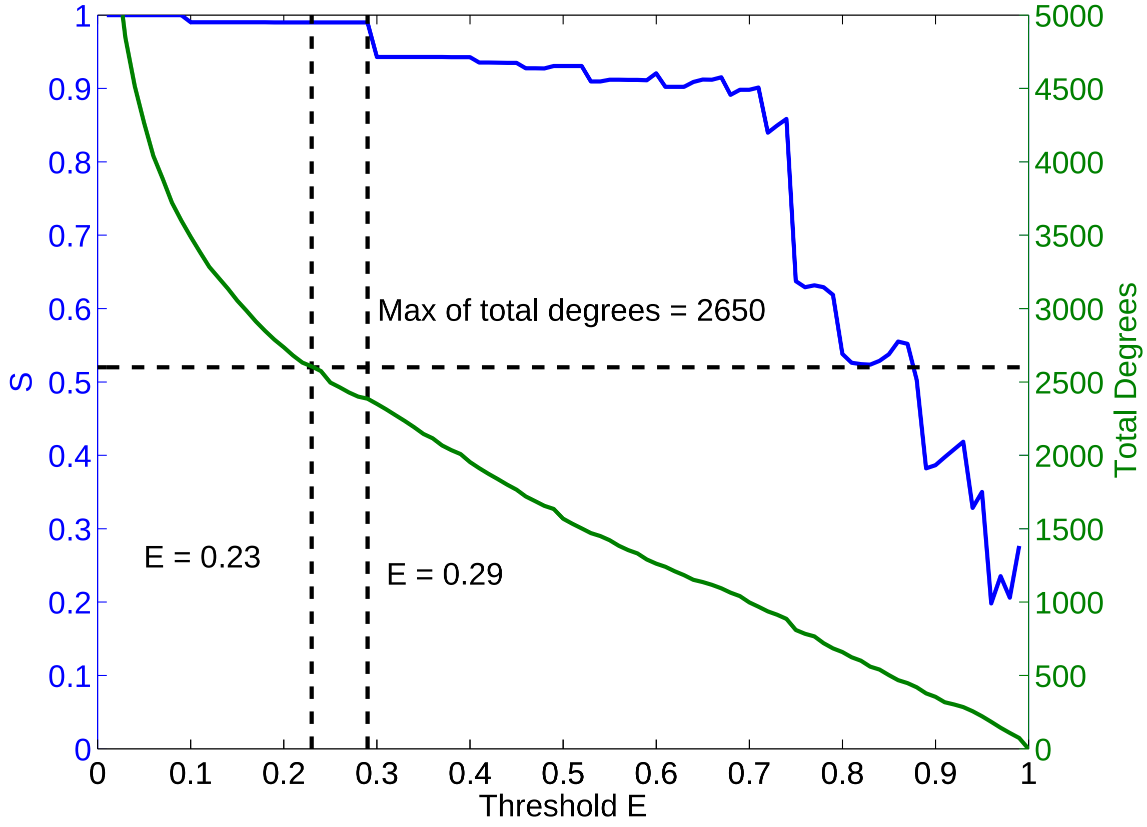

Figure 2: **Giant component size S and total degrees D_T of the idealized network constructed under different threshold E.** The blue curve represents S, while the green curve represents D_T. The right vertical dashed line represents the upper limit of $E = 0.29$ with S larger than 0.98. The right vertical dashed line represents the lower limit of $E = 0.23$ when D_T is smaller than 2650 (he horizontal dashed line).

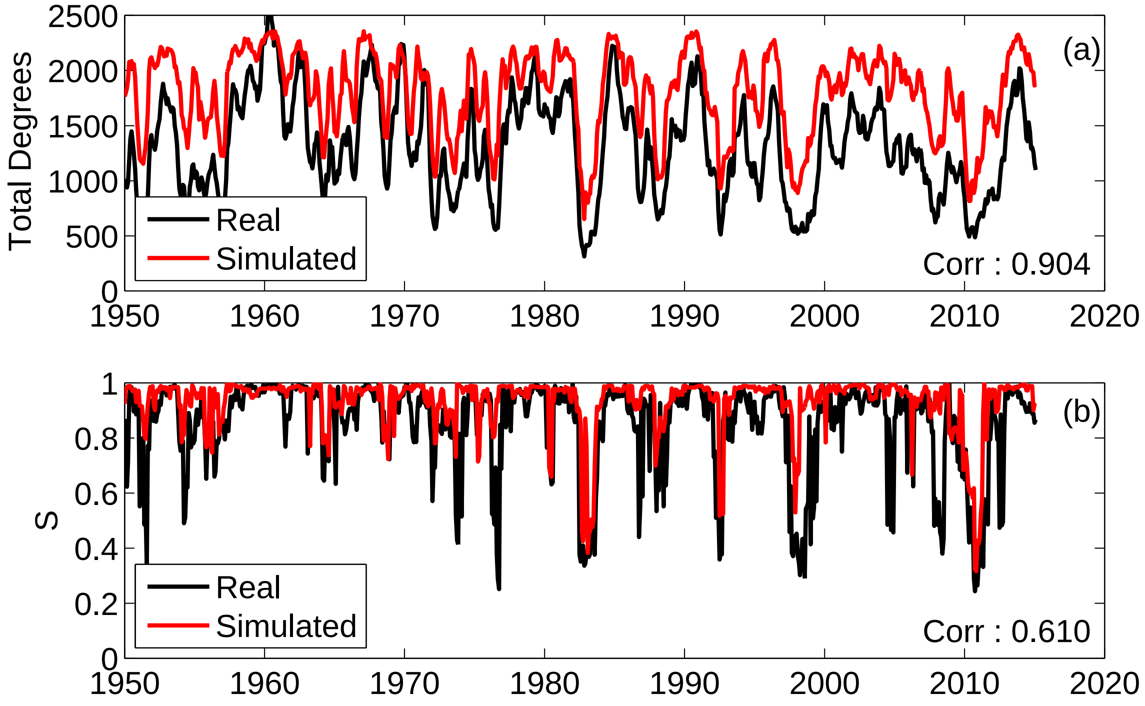

Figure 3: **Total degrees D_T and giant component size S of the real network and the idealized network.** a) shows the total degrees of connection D_T, and b) shows the giant component size S. The black curves are the results calculated from the real network, while the red curves are the results simulated from the idealized network. As one can see, the black and the red curves have similar patterns with very high correlation coefficient: 0.904 for D_T, 0.610 for S. Therefore, the idealized network indeed has the ability in simulating the reactions of real network to the attacks of El Niño/La Niña.
